# A Conversational Agent for Providing Personalized Preexposure Prophylaxis (PrEP) Support: Protocol for Chatbot Implementation and Evaluation

**DOI:** 10.2196/79966

**Published:** 2026-10-01

**Authors:** Fatima Sayed, Albert Park, Patrick Sean Sullivan, Yaorong Ge

**Affiliations:** 1 Department of Software and Information Systems, University of North Carolina at Charlotte, Charlotte, NC, United States; 2 School of Health Information Science, University of Victoria, PO Box 1700 STN CSC, Victoria, BC, V8W 2Y2, Canada, +1-250-721-8575; 3 Department of Epidemiology, Emory University, Atlanta, GA, United States

**Keywords:** HIV prevention, chatbots, conversational agents, large language models, retrieval-augmented generation, generative AI, PrEP, preexposure prophylaxis

## Abstract

**Background:**

Chatbots have the potential to reduce barriers to preexposure prophylaxis (PrEP), including lack of awareness, misconceptions, and stigma, by providing anonymous and continuous support. However, in the context of PrEP, chatbots are still nascent; they lack personalized informational expertise, peer experiential expertise, and human-like emotional support to facilitate future PrEP uptake. These personalized and relatable forms of support are crucial for increasing engagement, influencing health decisions, and fostering resilience and well-being.

**Objective:**

In this paper, we describe the iterative development and evaluation plans of a retrieval-augmented generation (RAG) chatbot for providing personalized information, peer experiential expertise, and human-like emotional support to PrEP candidates.

**Methods:**

We used an iterative design process consisting of 2 phases: prototype conceptualization and iterative chatbot development. In the conceptualization phase, we identified real-world PrEP needs and designed a functional dialogue flow diagram for PrEP support. Chatbot development included developing 2 components: a query preprocessor and a RAG module. The preprocessor uses the Segment Any Text (SAT) tool (developed by Markus Frohmann, Igor Sterner, Ivan Vulić, Benjamin Minixhofer, and Markus Schedl) for query segmentation and a Gemma 2 fine-tuned support classifier to identify informational, emotional, and contextual data from real-world queries. To implement the RAG module, we used Sentence-Bidirectional Encoder Representations from Transformers (SBERT) embeddings with cosine similarity, and performed topic matching to identify topically relevant documents based on the query topic to support document retrieval. Extensive prompt engineering was used to guide the large language model (LLM), Gemini-2.0-flash, in generating tailored responses. We conducted 10 rounds of internal evaluations to assess and improve the chatbot responses based on 10 criteria: clarity, accuracy, actionability, relevancy, information detail, tailored information, comprehensiveness, language suitability, tone, and empathy. Finally, we conducted a blinded comparative study and used a linear mixed model to validate the chatbot against a general LLM and real-world user responses.

**Results:**

We developed a RAG chatbot and iteratively refined it based on the internal evaluation feedback. Prompt engineering is essential in guiding the LLM to generate responses tailored to information, experiential, and emotional user needs. We found that prompt effectiveness varied with task complexity; this was likely due to LLM sensitivity to the structure of prompts and to linguistic variability. Prompt decomposition and segmenting prompt instructions helped improve comprehensiveness and relevancy for complex and long queries. The linear mixed model analysis revealed that while both the general LLM and RAG were preferred over user responses, the general LLM maintained a superior edge over the RAG chatbot across most criteria, with the exceptions of empathy and tone.

**Conclusions:**

Our RAG chatbot leverages social media data to provide personalized information, peer experiences, and human-like emotional support; these elements are essential for addressing PrEP misconceptions and promoting self-efficacy. Further analysis incorporating expert and user feedback will be conducted to help validate and improve the chatbot’s potential.

## Introduction

### Background

Increasing the uptake of preexposure prophylaxis (PrEP) [1-3] is a key strategy in the Ending the HIV Epidemic (EHE) initiative [4]. However, social and structural barriers to HIV treatment-seeking behaviors continue to impact health outcomes and drive inequities [5-7]. PrEP adoption has been hindered in the United States by lack of PrEP knowledge and prevailing anticipated and experienced stigma and discrimination [5,7-9]. Personal experiences shared by peers can improve the awareness [10,11], self-efficacy, and intrinsic motivation [12] of people considering PrEP use, mitigating their stigmatized beliefs [10], and informing health decisions [10,13]. People without financial means or ready access to providers experience limited access to health care [14,15]. Service deserts result in a lack of physically proximate service providers [6]; scarcity of services also disproportionately impacts those without financial means or access [16]. Equitable PrEP uptake requires facilitating access to PrEP resources while meeting the diverse needs of PrEP candidates [16]. Conversational agents (ie, chatbots) have the potential to provide anonymous, round-the-clock assistance in finding a PrEP provider, answering questions about PrEP, and providing emotional support. These services can help to overcome geographical and access barriers to seeking PrEP care.

Integrating chatbots into existing public health programs can significantly mitigate treatment barriers while enhancing user trust and adoption. However, to ensure scalability, deployment must account for infrastructure constraints and linguistic diversity. In low-resource settings where internet access, device affordability, and digital literacy may be limited [17], chatbots should be designed for local deployment to ensure data privacy and accessibility. This includes supporting offline functionality for mobile devices and using lightweight architectures to ensure accessibility on older hardware. Furthermore, to cater to cultural and behavioral preferences, it is essential to incorporate multilingual and multiplatform support. Providing such user-centric personalization is a vital step in reducing health disparities and ensuring that digital interventions are both accessible and equitable.

### HIV and PrEP Chatbots

Chatbots have demonstrated potential in disseminating HIV information [18,19], self-testing [18,20,21], access to and uptake of HIV prevention and care [22-24], and making positive behavioral changes relevant to HIV prevention and care (eg, self-disclosure of HIV status [25,26] and self-management of PrEP adherence [18,22,27]). AI technologies can be useful in developing and operating chatbot services, but their contribution is variable depending on the risk and complexity associated with health infrastructure. This is because risk and complexity influence the adoption of chatbots by users for improving health conditions [28]. Rule-based chatbots, which use scripted text based on simple rules (ie, keyword identification and pattern-matching techniques), are most commonly used to provide information [19,29] and support self-management of health behaviors (eg, setting up reminders) [22,25]. More advanced chatbots use information retrieval systems [23] and knowledge graphs [21] to help users identify HIV risk and navigate relevant information to mitigate barriers to PrEP uptake. For more complex needs such as identifying disorders and providing behavioral support (eg, for PrEP retention), chatbots use hybrid techniques that use AI tools (eg, machine learning [20,21,23] and natural language processing [21]) to learn patterns from previous conversations [30]. These approaches can also detect emotions [30] and perceive user characteristics based on past interactions [21], and can integrate dialogue rules for response generation. Two protocols for studies mention the use of a hybrid large language model (LLM; HumanX) chatbot for promoting PrEP uptake and use [24,27] and for providing HIV-related mental health support [22]. However, these studies do not specify details of the chatbot implementation. Such rule-based and hybrid health care chatbots are often criticized for their limited flexibility [31,32] and diversity of content [31-36], and insufficient contextual understanding [37,38] and personalization [23,39]. Such chatbots have also been reported to lack human-like emotional support [40], and this limits long-term user engagement and chatbot effectiveness [41]. LLMs, leveraging vast knowledge bases, can generate responses that are varied and context-relevant compared to rule-based and hybrid systems [42]. Beyond information retrieval, LLMs have demonstrated a significant capacity for cognitive empathy, the intellectual ability to identify and label a user’s emotional state and respond with supportive language [43,44]. However, researchers have noted that these responses often lack affective empathy [44], as the models lack human-like emotional experience and intrinsic motivation [43-45], leading to impersonal responses in sensitive and stigma-associated contexts [45]. This empathy gap suggests that while LLMs can extend general warmth, they struggle to provide the deep, personalized emotional connection found in human-to-human emotional support (ie, human-like emotional support).

To date, no study details the implementation of an LLM-based chatbot for providing various HIV and PrEP support, including emotional, informational, and peer experiential support to facilitate access to PrEP information and promote PrEP uptake and adherence. Although LLMs can offer improved personalization and human-like conversations [42,46], their performance can also be limited by their scope of specialized knowledge, diversity of available data, and the potential for LLM hallucinations [47,48]. Fine-tuned LLMs have been effective in performing domain-specific tasks; however, the small size of the training corpus limits their ability to generalize model capabilities across diverse user needs [31]. Prompt engineering [49] and retrieval-augmented generation (RAG) [48] techniques improve LLMs’ performance in terms of accuracy [50,51], relevancy [50,52], data privacy [53], and user personalization [54,55], which can enhance user trust in chatbot-provided support [56-58]. Using prompt engineering, LLMs can be guided to improve the precision and accuracy of generated responses [57]. RAG chatbots have indicated improved accuracy (34%) [49] and reduced hallucination (26.5%) [48] in generated responses compared to LLM question-answering bots. Compared to fine-tuned LLMs, RAG provides improved quality of responses in specialized domains by leveraging diverse external knowledge [27]. Using effective prompts to guide LLMs [32], RAG chatbots can offer greater personalization and relevance compared to rule-based and hybrid systems [27].

However, the development of chatbots based on LLM and RAG techniques is still nascent in the context of HIV and PrEP support and has not been implemented to address implicit user needs [48], answer long contextual queries [48], and generate personalized responses [59]. Addressing these issues in RAG chatbots can lead to providing accurate, flexible, personalized, and human-like responses to promote PrEP uptake.

Available literature [60-62] and our prior work have identified human-like emotional support components as essential elements to support behavior change for HIV medication taking and PrEP uptake. However, no prior work has developed RAG chatbots to provide personalized information and peer experiential expertise (eg, user stories) [63]. Existing HIV and PrEP chatbots focus on providing informational support [19,22,29] and emotional support according to therapeutic guidelines [27]. Effective support provided by humans relies on emotional support (ie, human-like emotional support) [64,65] and peer experiential expertise (ie, peer experiential support) [63,66], elements which are absent in existing health care chatbots. Providing effective human-like emotional support first requires understanding and communicating complex human emotional experiences [67-69]. The Willcox Feeling Wheel facilitates this process by providing a related vocabulary for 6 emotion categories [69]. To better reflect the real-world user queries that often seek practical user experiences (peer experiential expertise) [70], PrEP chatbots should provide peer experiential expertise support through pragmatic narratives of others in similar situations, which might enhance self-coping skills and emotional confidence, leading to improved quality of life [63,66]. Additionally, chatbots need to support back-and-forth dialogue exchanges, similar to real-world help-seeking behaviors, to facilitate clarification and understanding of complex concerns [71].

We develop a RAG chatbot that addresses the knowledge gaps in providing personalized PrEP support. We develop a support classifier to identify implicit PrEP user needs from long context queries by fine-tuning Google’s Gemma model. The RAG chatbot uses personalized prompts and an external knowledge base (facts and real-world PrEP experiences) to generate responses tailored to users’ informational, experiential, and emotional needs. To provide human-like emotional support, we use the Willcox Feeling Wheel to guide the chatbot in responding empathetically to the emotions expressed by the user. The chatbot also provides personalized peer experiential expertise support through pragmatic narratives of users in similar situations. We use extensive prompt engineering and fact validation techniques to improve accuracy, personalization, and transparency in generated responses.

### Protocol Goals

This protocol describes the development of a RAG chatbot in 2 phases. Phase 1 describes conceptualizing the chatbot architecture, and Phase 2 includes iterative development of the RAG chatbot to provide personalized information, peer experiential expertise, and human-like emotional support. Before prototype development, we conducted a separate study analyzing PrEP-related online social support exchanges to identify the social needs of PrEP candidates to conceptualize the chatbot architecture. Results of this study will be reported in a separate paper. We demonstrate the iterative development process focusing on prompt engineering, which is an integral part of the LLM RAG chatbot implementation. An exploratory user study was conducted to assess the quality of chatbot responses compared to real-world user responses and general LLM responses. We also discuss the implications of these findings, highlighting the feasibility of the chatbot prototype as a tool to facilitate future PrEP support.

## Methods

### RAG Architecture Overview

To improve accuracy and empathy, we designed an RAG architecture that explicitly separates informational, experiential expertise, and emotional support. Unlike a traditional LLM, which processes a user query based solely on its internal training data, this RAG framework uses a support classifier to identify specific user needs. The RAG chatbot then queries specialized knowledge bases to deliver tailored, evidence-based responses to the identified social support needs. This modular approach helps in controlling hallucination and improving the accuracy of the support provided. The mechanism involves segmenting user queries into specific needs and retrieving information from separate factual and experiential databases; the system ensures that responses are both evidence-based and empathetically aligned. The key features of the RAG chatbot include the following:

1. Modular retrieval: The system first segments and classifies user queries into 3 distinct categories—informational, experiential expertise, or emotional support. Using the enriched query segment, the RAG chatbot retrieves documents from specialized databases. It pulls from a clinical facts database for informational needs and an anonymized peer-experience database for experiential and emotional needs. This ensures that the chatbot only references verified data for facts and draws from real-world human narratives to provide empathetic and experiential support.
2. Contextual enrichment: A dedicated context manager handles query enrichment by ensuring sufficient information is present to respond to the query. If the query is underspecified, the manager prompts the user for additional context to refine the retrieval process.
3. Response generation: The retrieved context is integrated with specialized system prompts to generate the final response. During this stage, the system leverages a human emotional framework to map the user’s expressed feelings to a matching emotional vocabulary. This approach facilitates nuanced emotional support that aligns with the user’s specific emotional state and is grounded in real-world human empathy. To maintain cultural authenticity and relatability, the system incorporates direct quotes and real-world narratives from the experience database, providing responses grounded in genuine human experience rather than synthetic advice.

### Phase I: Prototype Conceptualization

To inform the goals of our RAG chatbot prototype, we analyzed 3020 publicly available Reddit conversations focused on PrEP from April 2011 to March 2024. We focused on the informational (including peer experiential expertise) and emotional (ie, psychosocial) support because these are the 2 main types of support that are exchanged in this forum and because these have the potential to impact broader behavioral trends related to PrEP use [72]. Our database for RAG aligns with the previous literature [73]. We found that more than 88% (2658/3020) of user PrEP needs included informational and emotional need items. Informational needs centered on PrEP facts and related experiences, and emotional needs focused on reassurance, HIV and PrEP concerns sharing, empathy, and sympathy, based on the psychosocial constructs of Social Support Behavior Code (SSBC) [74,75]. PrEP candidates’ emotions can be described with the Willcox Feeling Wheel [69], including sad, mad, scared, joyful, peaceful, and powerful.

Real-world user queries were multifaceted, consisting of multiple informational and emotional needs. Chatbots must (1) preprocess each input query into semantic segments, (2) generate responses for each segment, and (3) combine them into a final response. Based on literature [73] and our prior work, our chatbot was designed to (1) generate personalized responses, (2) provide accurate and comprehensive information to user queries, (3) provide peer experiential expertise support, and (4) respond empathetically to the emotions expressed by the user. Functional dialogue flow diagrams were created (Figure 1) to visually communicate with our team members and developers how the chatbot would achieve these aims.

To identify user needs, we created a PrEP-related dataset. This dataset was annotated with 3 categories: informational (including facts and peer experiential expertise), emotional, and context. Subsequently, we built a classifier to parse user inputs and categorize them into the 3 categories. The classifier further classified informational needs into facts and peer experiential expertise. For informational needs, we specifically designed the chatbot to provide information (eg, facts and actionable advice) and peer experiential expertise support (ie, other people’s experiences and opinions). For emotional needs, we designed the chatbot to identify user emotions based on the Willcox Feeling Wheel [69] and to generate a suitable response using relevant SSBC psychosocial constructs known to impact PrEP uptake (eg, reassurance, understanding, empathy, sympathy, validation, relief of blame, and compliment) [60,76].

**Figure 1.**
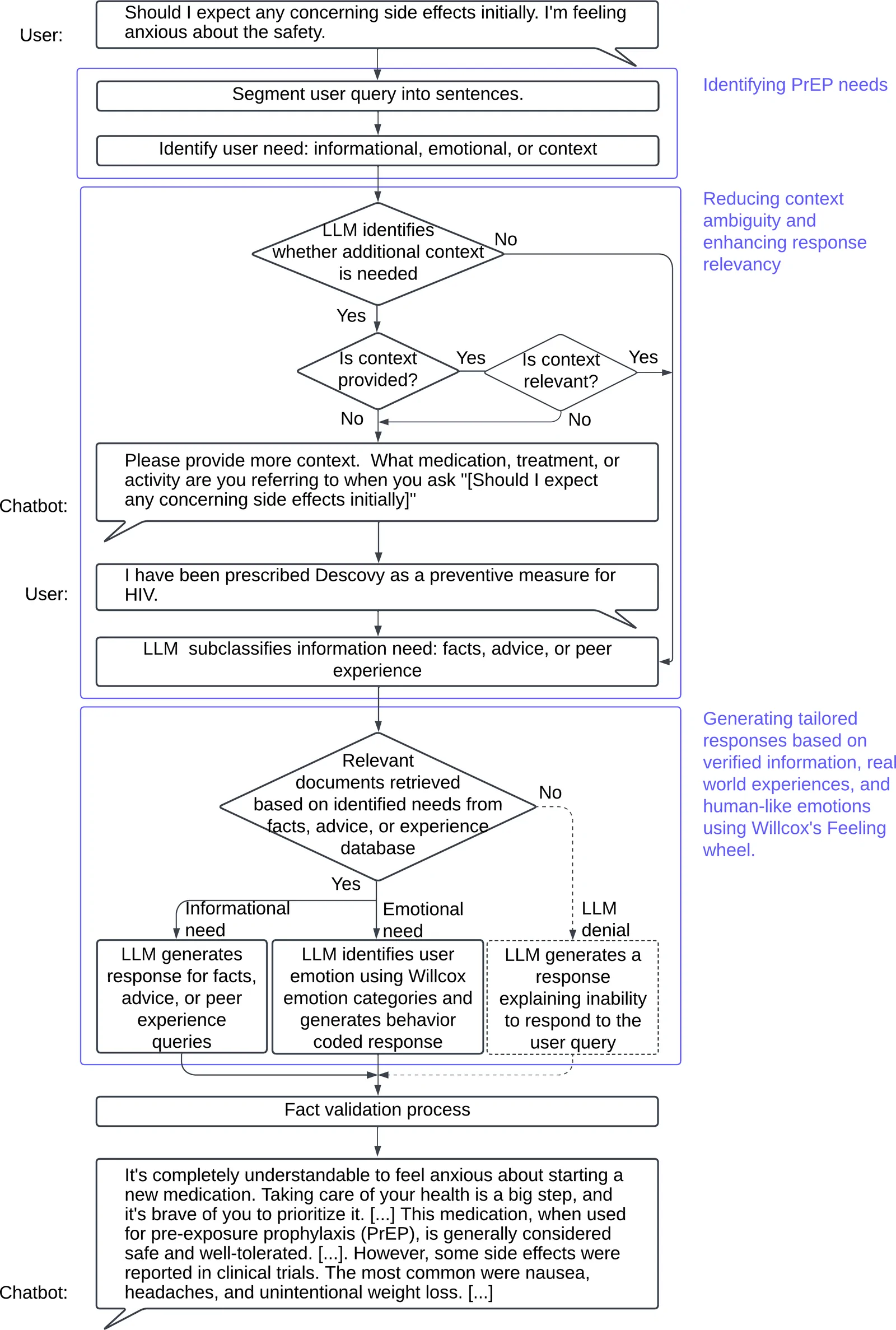
An example of a generated response using a paraphrased real-world query from Reddit. The retrieval-augmented generation process includes (1) semantically segmenting the query into sentences and classifying each segment into 1 of the 3 categories: “information,” “context,” or “emotion”; (2) checking for additional context; (3) retrieving the top 5 relevant documents from specific databases; (4) identifying user emotions from emotional segments and retrieving documents via topic matching; and (5) generating a response using personalized prompts. The dotted line in the diagram indicates ongoing updates. LLM: large language model; PrEP: preexposure prophylaxis.

### Phase II: Iterative Prototype Development

#### Overview

The iterative development cycle (Figure 2) consists of four processes: (1) system architecture design—drafting initial prompts for guiding LLM response generation, (2) internal evaluation of chatbot responses, (3) modification of the RAG chatbot based on internal evaluation feedback, (4) exploratory user evaluation to compare the RAG chatbot responses against general LLM and real-world user responses, and (5) a heuristic evaluation by HIV experts and a large-scale user study to validate accuracy, safety, ethical alignment, and user trust of chatbot responses prior to real-world deployment.

**Figure 2.**
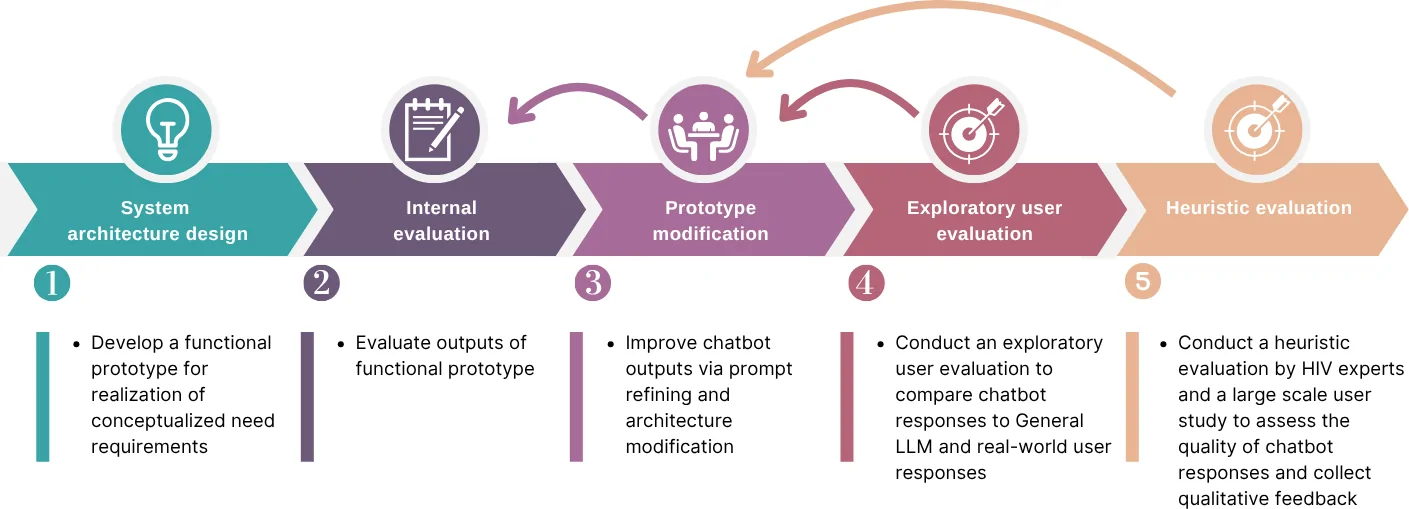
Iterative development and refinement process of the retrieval-augmented generation chatbot for providing support to preexposure prophylaxis candidates. The process initiated with (1) system architecture design, followed by (2) internal evaluation involving developers and an HIV expert to assess functional requirements. This resulted in a recursive cycle of (3) prototype modification, in which the retrieval-augmented generation chatbot is modified based on internal evaluation and findings from (4) exploratory user study and future (5) heuristic evaluation and large-scale user study. LLM: large language model.

#### System Architecture

##### Overview

The RAG chatbot was developed using Python programming and by accessing the Google Gemini API. The chatbot consists of 2 main modules (Figure 3): a preprocessor and a RAG module. The RAG, in turn, leverages 2 databases: a facts database and an experience database. The facts database contains verified information from factsheets, brochures, and websites on HIV and PrEP from public health agencies and private health organizations. The experience database contains Reddit user experiences and emotions related to PrEP concerns. The preprocessor extracts subqueries and context information from user input. To enhance model determinism and minimize irrelevant generation, the information retriever uses the extracted information to handle context management and information subclassification to enhance semantic retrieval and output relevant documents. Finally, the LLM generator synthesizes the retrieved documents and user context through personalized prompt engineering, providing a robust framework for aligning model behavior with user-specific needs while maintaining high factual fidelity.

**Figure 3.**
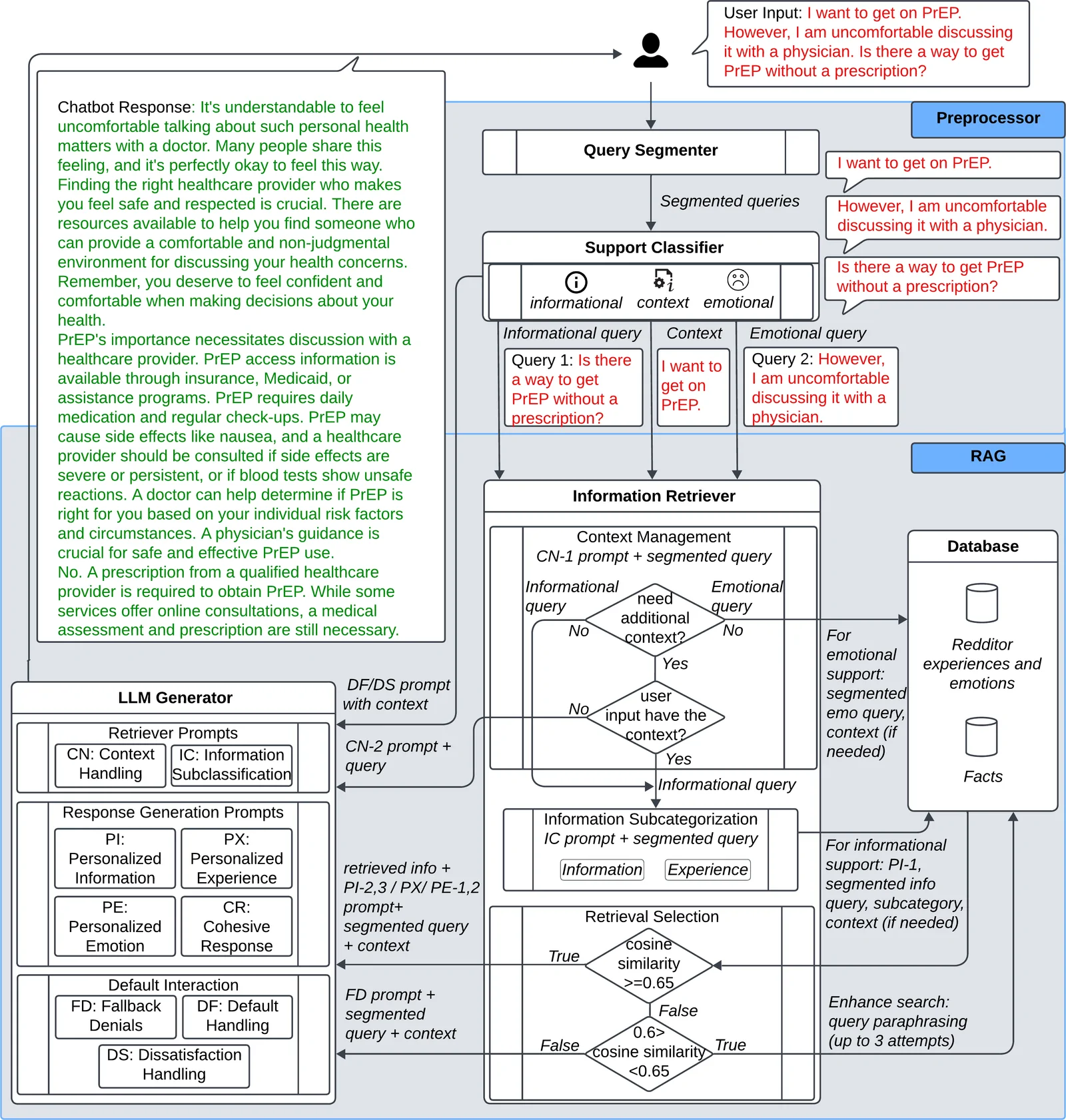
Architecture of the retrieval-augmented generation chatbot used to generate personalized information, peer experiential expertise, and human-like emotional responses for providing tailored responses. LLM: large language model; PrEP: preexposure prophylaxis; RAG: retrieval-augmented generation.

##### Preprocessor Module

The preprocessor module consists of 2 components: a query segmenter and a support classifier.

###### Query Segmenter

The query segmenter semantically segments a user input into sentences using the SAT tool [77]. The tool is highly robust because it is minimally reliant on punctuation and space characters. This makes it an optimal choice for handling diverse writing styles and complex real-world user queries. Each segment is then passed to the support classifier.

###### Support Classifier

####### Overview

The support classifier was used to classify the intent of the input query segments to guide the retrieval process. For segments identified as informational, a few-shot information subclassifier served as a routing layer: it directed experience-based queries to the experiential database and fact-based queries to the factual database. This classification-driven routing ensures that the retrieval pipeline is grounded in the appropriate knowledge database. A detailed description of this routing logic and how classifier labels trigger specific database retrieval is provided in the “Information Subcategorization” section.

####### Dataset

We created the PrEP dataset, which consists of semantic sentences extracted from online user PrEP discussions and manually labeled them into 1 of the 3 categories—informational, emotional, and context. For fine-tuning, we used 2 batches of data: the entire (n=32,081) PrEP dataset (class-unbalanced) and a class-balanced subset (n=10,041) of the PrEP dataset.

####### Classifier Fine-Tuning

We developed 6 support classifiers by fine-tuning each batch of data using 3 models: Bidirectional Encoder Representations from Transformers (BERT)-base-uncased model, Gemma 2B-it [78], and Gemma 2 2B-it [78]. Gemma model series is lightweight and hardware-flexible, offering low latency and balancing performance and efficiency, making it suitable for chatbot deployments [78]. BERT is the state-of-the-art model used in text classification tasks.

We used Parameter-Efficient Fine-Tuning (PEFT) with standard Low-Rank Adaptation (LoRA) configurations (α=32 and rank=64) [79] on a sequential classification task for fine-tuning the Gemma models. Based on previous literature on LLM fine-tuning performance, we fine-tuned the Gemma 2B-it model for 5 epochs with an early stopping patience of 2 epochs [80,81]. Similarly, the BERT model was fine-tuned for 5 epochs.

####### Evaluation Metrics

We used *F*_1_-score and accuracy to compare the performance of the 6 classifiers on the class-balanced (macro average) and class-unbalanced (weighted average) datasets.

### RAG Module

The RAG module consists of an information retriever and an LLM generator.

#### Information Retriever

The information retrieval process includes context management, information subcategorization, and retrieval selection.

##### Context Management

The context handler instructs the LLM (Gemini-2.0-flash) using the context handling prompt 1 (CN-1) to determine whether queries require additional context. If no additional context is needed, only the user query is used for retrieving relevant documents. If additional context is needed, our chatbot will ask users for additional context using the context handling prompt 2 (CN-2). With additional context provided by the user, the chatbot then retrieves relevant documents using both the context and user query.

##### Information Subcategorization

To generate personalized responses, the information needs were further subcategorized into (1) seeking facts or (2) requests for peer experiential expertise, using the information subclassification few-shot prompt (IC; Table S1 in Multimedia Appendix 1). The identified information subcategory (ie, facts or peer experiential expertise) was used to guide the retrieval process. For queries seeking facts, documents were retrieved from the facts database, and for queries seeking peer experiential expertise, documents were retrieved from the experience database. We included the “advice” subclassification in the few-shot examples as ongoing updates include integrating the “MedHelp” database, an online health discussion board containing expert advice related to PrEP.

##### Retrieval Selection

The retriever uses Sentence-Bidirectional Encoder Representations from Transformers (SBERT) embeddings [82] to retrieve relevant documents from the database. We used SBERT embeddings due to their bidirectional contextual understanding [82], computational efficiency [82], and superior performance in identifying semantically similar sentences, coupled with synonyms and negation lexical variations [83], which are better suited to cover various user writing styles. The retriever uses cosine similarity, and the LLM identifies topics for information retrieval; this was done to provide facts for informational or emotional support. We used topic matching to enhance the retrieval of a wide variety of relevant documents from the facts database, including context-rich queries that challenge semantic retrieval [84] in short query databases. We manually reviewed the facts database to identify 47 broad HIV-PrEP–related topics (Textbox 1). To annotate the facts database, we instructed the LLM (Gemini-1.5-flash) to assign the most suitable topic to each query from this list. If no relevant topic is identified, we instructed the LLM to generate a closely relevant topic to have the ability to adapt to unforeseen user needs. We then manually reviewed the annotated topics to include any LLM-generated new topics in the topic list and to merge semantic duplicates. We used Gemini-1.5-flash, the latest model at that time, and retained the topic lists because we manually verified all the LLM-generated topics and were satisfied with the results.

Textbox 1. **List of topics used in retrieval-augmented generation topic document retrieval for factual support. The HIV-PrEP (pre-exposure prophylaxis) topic list was created by manually reviewing the facts database and updated using large language model topic assignment. Topic document retrieval from the facts database was used to improve sensitivity in unstructured queries.**

(1) PrEP; (2) PrEP eligibility; (3) PrEP effectiveness; (4) PrEP access and cost in Australia; (5) PrEP access and cost; (6) HIV testing, (7) HIV self-test; (8) postexposure prophylaxis (PEP); (9) PrEP administration and dosage; (10) apreptude or injectable PrEP; (11) PrEP side effects; (12) HIV and AIDS; (13) risk and transmission; (14) HIV prevention strategies; (15) safety and precautions; (16) cabotegravir; (17) sexually transmitted infections (STIs); (18) viral load or undetectable or untransmissible; (19) HIV treatment; (20) PrEP medications; (21) emtricitabine or tenofovir disoproxil fumarate; (22) emtricitabine or tenofovir disoproxil fumarate administration; (23) emtricitabine or tenofovir disoproxil fumarate side effects; (24) drug interactions; (25) emtricitabine or tenofovir disoproxil fumarate eligibility; (26) antiretroviral or HIV drug side effects; (27) test disclosure and anonymity; (28) stigma and its impact on people living with HIV; (29) PEP access and cost; (30) PEP side effects; (31) Apretude or injectable PrEP side effects; (32) cabotegravir side effects; (33) PEP eligibility; (34) PrEP access and cost in the United Kingdom; (35) medication adherence; (36) Truvada side effects; (37) healthy lifestyle; (38) Descovy; (39) PrEP safety and precautions; (40) HIV prevention strategies; (41) Truvada; (42) PrEP eligibility in the United Kingdom; (43) Descovy side effects; (44) HIV or AIDS drugs; (45) Truvada access and cost; (46) Descovy access and cost; and (47) emtricitabine or tenofovir disoproxil fumarate safety and precautions.

##### Retrieval for Factual Support

For factual support, we retrieved the top 5 documents from the facts database with a cosine similarity score ≥0.65. The cosine similarity thresholds were determined based on experiments, and we set k=5 for document retrieval based on previous evaluation benchmarks [85,86]. In addition, we selected 5 topically relevant documents, using the topic identified from the 46 HIV-PrEP topics (Textbox 1). To identify the topic of an informational query, we instructed the LLM (Gemini-2.0-flash) using the HIV-PrEP topic list and the personalized information prompt-1 (PI-1) to assign one suitable topic to the user query. Retrieving documents using cosine similarity was based on the context need. If context is needed, we retrieved 3 sets of top 5 relevant documents (Figure 4) by comparing cosine similarity between (1) the context-added query and database questions (m1), (2) the context-added query and database answers (m2), and (3) the context-free query and database questions (m3; Figure 4). Both database questions and answers were semantically searched to improve the retrieval precision. Cosine similarity between the context-free query and database answers did not improve the retrieval precision and was therefore not used in processing the context-free queries. As the majority of the facts database consists of short questions from PrEP factsheets and brochures, the retriever often failed to retrieve relevant information for queries with additional context. If no context is needed, we retrieved the top 5 documents by only comparing cosine similarity between the context-free query and the database questions.

**Figure 4.**
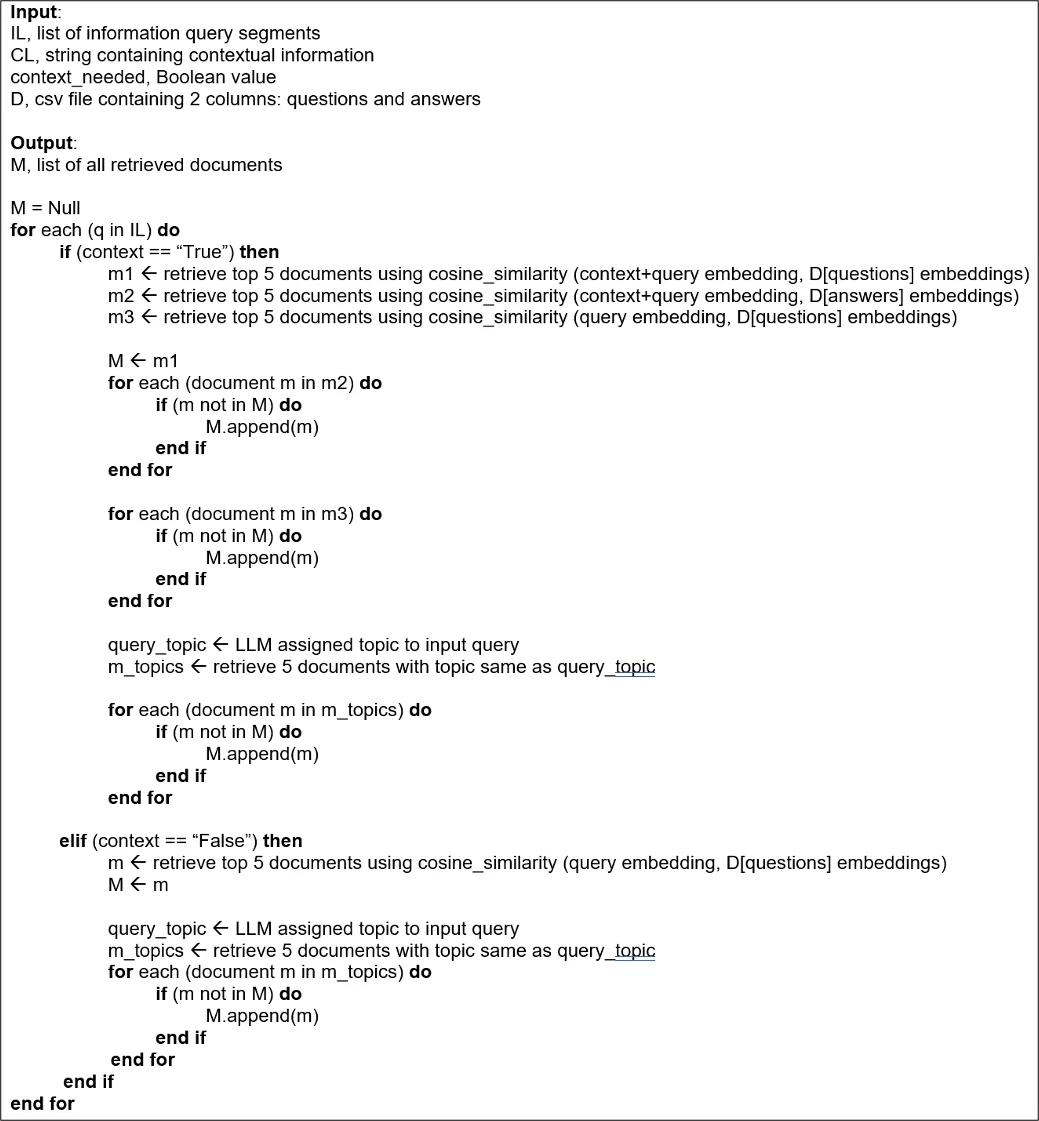
Retrieval algorithm pseudocode for improving retrieval precision in factual queries using cosine similarity.

##### Retrieval for Peer Experiential Expertise and Emotional Support

For peer experiential expertise support, we retrieved the top 5 documents from the experience database using cosine similarity with a score of at least 0.65. When context is needed, we separately used both the query and context for retrieval to overcome context-dependent retrieval failure. For emotional queries, the top 5 similar documents were retrieved from the experience database in addition to providing relevant facts. Similar to document retrieval for peer experiential expertise support, we retrieved the top 5 documents from the experience database using cosine similarity with a score of at least 0.65 and, when context is needed, used both the query and context for retrieval. As information can be helpful in addressing certain HIV-PrEP emotional concerns [87], we retrieved facts relevant to the emotional query.

To improve the sensitivity, we also used up to 3 paraphrase queries with a lower cosine similarity score, between 0.6 and 0.65. For paraphrasing, we used the T5 paraphrase generator from the Transformers Hugging Face library [88] for its demonstrated superior performance over GPT-3 and ChatGPT in capturing semantic relevance between sentences [89].

### LLM Generator

Once all the necessary data were retrieved, we instructed the LLM (Gemini-2.0-flash) to generate responses using the retrieved information. This process was guided by personalized prompts (PI [personalized information], PE [personalized emotion], and PX [personalized experience]) and the user need (ie, informational, peer experiential expertise, and emotional responses) as described in Table S1 in Multimedia Appendix 1. We iteratively refined the prompts via prompt engineering to improve comprehensiveness, accuracy, relevancy, transparency, and response coherence. The PI-2 prompt instructs the LLM to provide a detailed response, followed by the PI-3 prompt to check the consistency of factual queries. For peer experiential expertise queries, we used the PX prompt that instructs the LLM to use the retrieved documents and generate a third-person narrative with explicit statements of partner conversations and direct quotes, when possible, to emphasize transparency.

For providing human-like emotional support, we used PE-1 to first identify the feelings based on the 6 Willcox feeling categories and to generate a suitable response reflecting SSBC psychosocial constructs (ie, sympathy, understanding, empathy, relief of blame, validation, compliment, or reassurance) for the identified feeling. Identifying user feelings allows responding with suitable emotional expressions that are typically associated with human responses. We also found that general LLM responses to emotional queries lacked deeper understanding of underlying emotions and human-like emotional behaviors, consistent with findings in previous literature [90]. The RAG LLM was instructed to use the style and tone from the retrieved emotional documents, which comprise peer-to-peer emotional support. When no relevant documents were retrieved for an emotional query, we used the PE-2 to generate a suitable response reflecting SSBC psychosocial constructs for the identified user feeling, without using the style and tone from the retrieved documents. Fact responses were validated by the fact validator. Then, the cohesive response (CR) prompt combines all subquery responses, instructing the LLM to smooth the information flow into a single CR. Finally, the LLM provides a combined response.

### Fact Validator

Responses providing factual information were validated with the documents from the facts database, a step toward eliminating LLM hallucination. Our fact validation algorithm consists of 2 steps: fact identification and fact validation. To reduce overreliance on LLMs, we used a deterministic technique to identify semantically relevant facts from the retrieved documents (ie, the same top 5 documents retrieved from the information retriever process). This is done to address a known issue: that LLMs often provide imperfect fact identification when they break down complex information [91,92]. To identify the most relevant facts for validation, we (1) segmented the generated response and retrieved documents using the SAT tool and then (2) extracted the most relevant fact-supporting document segment (step 1) based on SBERT and cosine similarity (ie, highest similarity score). This creates pairs of generated response segments that correspond to their fact-supporting document segments. The LLM was instructed to use the response-document pairs and the FV-1 prompt (step 2) to identify any response segments that are not supported by the document segments identified with cosine similarity. Providing the subset of relevant fact segments to the LLM narrows its focus, improving validation accuracy. Then, using any generated response segments with inaccurate facts and the FV-2 prompt, the LLM was instructed to extract supporting facts from the retrieved documents (step 3).

To evaluate the performance of the fact validator, we manually verified the performance for 1033 fact response segments from 100 generated responses for randomly sampled query responses that the LLM generated. The fact validator achieved a 97.39% validation accuracy based on 1006 sample items. The accuracy was calculated as the percentage of fact response segments that were fully supported by the facts database, as determined by the research team. Only 3% (27/1033) of the generated response segments were not able to be validated with the facts database (95% CI 1.85‐3.88). These included response segments providing general information (n=12, eg, *“*Other side effects [of the medication] include changes in the immune system, dizziness, diarrhea, nausea, vomiting, headache, rash, and gas*.*”) and personalized information (n=8, eg, “Given the recent unprotected anal sex with someone who recently learned they had syphilis, your friend should get tested for STIs as soon as possible.”). We also found false negatives (n=7), where the LLM extracted facts that could not support the generated response segment in step 3. For example, when validating a response segment (eg, “Truvada alone is not sufficient for post-exposure prophylaxis (PEP).”) with an accurate and semantically retrieved document segment*,* the LLM extracted facts that could support the generated response segment (eg, “It is worth noting that Truvada must be used correctly (without skipping doses) whether it is used in combination with other ARVs in the control of HIV, or for PrEP.”). Based on these data, we opted for a 3-step validation process because the semantic retrieval (step 1) identified over 82.10% (848/1033) of accurate response segments, whereas the LLM identified (step 2 and step 3) 15.30% (158/1033) of accurate response segments. It is also important to note that we also found that LLM-based validation was less consistent compared to the deterministic techniques. For instance, when we processed the same sets of 100 samples 10 times, the LLM changed its final validation responses (step 2 and step 3) in 44% of samples. It is important to note that the 44% does not indicate that earlier responses were inaccurate. Rather, the nondeterministic nature of LLMs (as probabilistic models) relying solely on LLMs can hinder the validation process. This highlights the need for mixed validation approaches, combining deterministic techniques and LLMs to eliminate LLM hallucinations.

### Internal Evaluation of the Chatbot Responses and Prompt Refinement

#### Internal Evaluation

The RAG chatbot was evaluated to assess the functional requirements following the iterative evaluation design process [93], with a focus on (1) accurately identifying user informational and emotional needs, (2) providing relevant and accurate information, and (3) generating personalized and emotionally suited responses. Developers conducted 10 rounds of internal evaluation of chatbot responses. Paraphrased user inputs from the experience database were used to create 2 test queries for each support need (ie, informational, peer experiential expertise, and emotional). In each of the 10 evaluation rounds, we selected (1) one simple and direct query and (2) one complex and context-dependent query for each support need. Responses were generated for the 6 test queries per round, which were then iteratively reviewed in 10 rounds of internal evaluation (n=60) by at least 2 chatbot developers and at least 1 HIV expert. This qualitative evaluation of 60 total responses helped to identify inaccuracies and refine the LLM prompts and was followed by chatbot modification to address issues identified by the reviewers. The most frequently occurring issues were as follows: (1) low retrieval sensitivity and precision for context-dependent queries, (2) contradicting information from user discussions, (3) response generation using inherent knowledge, (4) irrelevant response context, (5) chatbot fictional narratives, (6) incomprehensive responses, and (7) noisy text (eg, unwanted phrases and special characters). Generated responses were evaluated based on the following 10 criteria: clarity [94,95], accuracy [89,94,96], actionability [96], relevancy [89,94], information detail [95], tailored information [89,94], comprehensiveness (questions answered fully) [95], language suitability [94], tone [95], and empathy [89]. The responses were also manually reviewed to verify whether the chatbot generates responses only using inherent knowledge (ie, verifying whether generated content is outside the scope of retrieved documents).

#### Prompt Engineering

##### Overview

First, we created level-1 prompts (ie, PI, PX, PE, and CR) that were simple and direct instructions for generating a suitable response to a user query [97]. The responses generated using level-1 prompts were then internally evaluated to assess functional requirements. Initially, the LLM rarely generated inaccurate information based on the user experience database. After we finalized the RAG database architecture, we followed LLM prompt engineering techniques [49,97]. Prompts were iteratively refined following 10 rounds of internal evaluation to result in the final level-2 prompts (ie, PI, PX, PE, and CR) that were more specific and structured to enhance information comprehensiveness, accuracy, relevancy, transparency, and response coherence.

All prompts were checked sequentially for overlapping effects. The process started with information subclassification (IC) and context handling prompts (CN-1 and CN-2). Then, we implemented personalized information prompts, followed by prompts for topic identification (PI-1), personalized information response generation (PI-2), and verifying relevancy (PI-3). Then, the PX and personalized emotion prompts (PE-1 and PE-2) were developed. Finally, we developed and refined the prompt for response cohesion (CR). The final prompts are shown in Table S1 in Multimedia Appendix 1.

##### Prompt Engineering for Information Comprehensiveness

To improve information comprehensiveness and low retrieval sensitivity and precision, we refined the level-1 PI-2 prompt by instructing the LLM to segment the user query and provide a detailed response to all segments using the retrieved documents. In contrast, our initial level-1 PI-2 prompt (Table S2 in Multimedia Appendix 1) simply instructed the LLM to provide a response to a user query using the retrieved documents; this generated responses that were not comprehensive (ie, the question was not answered fully).

##### Prompts for Response Accuracy

We refined our level-1 IC, CN-1, PI-2, PE-1, FV-1, FV-2, and PX prompts (Table S2 in Multimedia Appendix 1) to improve the accuracy of generated responses. These prompt refinements helped address issues related to low retrieval sensitivity and precision, contradictory information, response generation using inherent knowledge, and fictional narratives. We refined the zero-shot IC prompt to a few-shot IC prompt to improve the accuracy of subclassifying informational needs. To improve context handling, the level-1 CN-1 prompt was iteratively paraphrased to verify whether additional context is needed for queries.

To enhance the accuracy of generated information, we refined the PI-2, FV-1, FV-2, and PX prompts to emphasize (by using “double asterisk” in the LLM prompts) the use of only the retrieved documents for generating a response. Using the level-1 PI-2 prompt, the LLM still relied on inherent knowledge in responding to factual queries, even when instructed to use the retrieved factual documents to generate a response. The level-1 FV-1 and FV-2 prompts failed to identify facts that supported generated response segments. This was primarily due to a lack of clarity in defining factual consistency. This led to false positives, in which the LLM identified facts that only partially support the response segments. To prevent generating fictional real-world experiences, we refined the level-1 PX prompt, which instructed the LLM to clearly indicate other people’s experiences or opinions as a third-person narrative using the documents retrieved from the experience database.

To avoid inaccurate but anecdotal information in the experience database, we refined the PE-1 prompt to instruct the LLM to avoid using facts retrieved from the experience database to provide factual support. Although the level-1 PE-1 prompt included an instruction to use the retrieved documents only to reflect style and emotional tone, the LLM still generated factual support using the experience database, which was identified when a response contained some inaccurate opinions retrieved from the experience database. For example, the experience database contained a user post “...The risk, even if he is undetectable, isn't zero, but it’s pretty low...,” which contradicts the recognized medical information [98].

##### Prompts for Information Relevance

To strengthen information relevance, we developed the PI-3 prompt (refer to Table S1 in Multimedia Appendix 1 for the final refined prompt) that verified the consistency of generated responses with the user query. The level-1 PI-2 prompt generated responses that lacked contextual awareness, specifically regarding locations or populations. Refining the PI-2 prompt to have the additional task of verifying response consistency proved ineffective. This was a consistent theme in prompt engineering. To reduce the complexity of prompting, we developed a separate PI-3 prompt to verify consistency after generating a response using the PI-2 prompt.

##### Prompts for Information Transparency

To prevent generation of fictional narratives, we refined the PX prompt by instructing the LLM to include direct quotes of the user’s personal experiences when available and requiring the LLM to explicitly state that the response information was based on other people’s experiences (eg, “... This response is based on other people’s experience.”).

##### Prompts for Response Coherence

To improve readability, comprehension, and user experience, we refined the PI-1, PI-2, PE-1, and PE-2 prompts to remove noisy responses, specifically unnecessary special characters (eg, * in “**Cost of PrEP in the US:**...” and [ in “[PrEP provided...infection.]”) and logical reasoning, which included chatbot-provided explanations for prompt execution (eg, “Okay, here are the revised responses, aiming for smoother flow and a more natural conversational tone: ...”) in responses.

### Exploratory User Evaluation

#### Study Design and Recruitment

An exploratory blinded-comparative online user study was conducted to evaluate the RAG chatbot’s output performance, where participants were presented with 3 sets of responses for each query: a real-world user response, a RAG-generated response, and a general LLM baseline (Gemini-2.0-flash). This study serves as an exploratory content validation and protocol design phase, establishing the foundational functional readiness of the system as well as future user studies. A total of 7 participants were recruited through personal networks and students at the authors’ institution.

#### Data Sampling

A batch of 25 queries and corresponding user responses were randomly sampled from the experience dataset (Reddit). For each query, three response types were compared: (1) user response: real-world user response from Reddit, (2) RAG chatbot response: responses generated by the proposed RAG chatbot, and (3) general LLM: responses from the Gemini-2.0-flash model (temp=0.65).

The evaluation was delivered via structured online questionnaires (Google Forms). Each participant evaluated a randomly selected subset of 15 queries. To ensure internal validity, the 3 response types were shuffled and deidentified for every query, ensuring participants rated the content quality without knowledge of the response source.

### Measures

Two types of questionnaires were developed for the study using Google Forms. The demographic questionnaire collected participant information on demographic and professional experience. The questionnaire consists of 6 deidentified questions asking participants about their age, gender, ethnicity, race, region, and professional background.

The chatbot response evaluation questionnaire was used to assess the quality of the responses based on 10 criteria (Table 1): information accuracy [89,94,96], information clarity [94,95], information actionability [96], information detail [95], relevance [89,94], tailored information [89,94], comprehensiveness-question answered fully [95], suitable tone [95], empathy [89], and suitable language [94]. The questionnaire contained instructions and definitions of each criterion along with the set of PrEP queries and corresponding responses. Participants were required to rate each response on a 5-point Likert scale for all criteria. Based on prior literature on expert-reviewed chatbot responses [99] suggesting good to excellent performance, we determined a response quality rating of above 4 on a 5-point scale for each of the 10 evaluation criteria.

**Table 1.** Evaluation criteria and descriptions provided in the chatbot response evaluation questionnaire.

Attribute and criterion	Description
Information quality	
Information accuracy [89,94,96]	Is the information provided factually correct and free from misinformation?
Information clarity [94,95]	Is the information presented in a clear, concise, and easy-to-understand manner?
Information actionability [96]	Does the response provide practical, actionable advice that the user can implement?
Information detail [95]	Does the response provide sufficient detail?
Response relevance and appropriateness	
Relevance [89,94]	Does the response directly address the user’s question and concerns?
Tailored information [89,94]	Is the information provided tailored to the specific needs and circumstances of the user?
Comprehensiveness: question answered fully [95]	Does the response fully answer the question without leaving significant gaps or requiring further clarification?
Language and tone	
Suitable tone [95]	Is the tone of the response appropriate for the context and the user’s overall emotional state?
Respectful, empathetic, and considerate manner [89]	Does the response demonstrate empathy and understanding for the user’s situation? Is the response respectful and non-judgmental?
Suitable language [94]	Is the language free from jargon or technical terms that may be unfamiliar to the user?

### Statistical Analysis

To account for the nonindependence of observations where multiple ratings were provided by the same participants, a linear mixed-effects model was used using the *lme4* package in R (v4.3.1; R Core Team). With 7 participants evaluating 15 queries across 3 response types and 10 evaluation criteria, the model analyzed a total of 3150 response evaluations. Participants and queries were treated as random effects to control individual rater bias and item variability. Quantitative analysis was conducted using R (R Core Team) and Microsoft Excel.

### Phase III: Planned Prototype Enhancement and Heuristic Evaluation

#### Future Prototype Enhancements

To further improve the quality and diversity of data, we will expand the data sources to include 2 additional discussion boards from MedHelp and POZ community (HIV positive) to broaden the knowledge base. MedHelp and POZ databases consist of PrEP-related conversational dialogues among community members. We will develop prompts specifically tailored for varying formats and structures [100], including conversational dialogues, question-answer pairs, and dialogues with quotes. We will update the chatbot architecture to maintain historical dialogues to reflect real-world multiturn conversations and include denial capabilities to set clear expectations for users and ensure safety compliance. We will also improve the fact validator’s accuracy to reduce the impact of LLM’s inherent inconsistencies when extracting facts to support generated responses. The final verification will focus on the accuracy of the responses rather than on the fact validator’s performance using the retrieved documents.

#### Planned Heuristic Evaluation: Expert Evaluation

To assess chatbot response quality, an iterative heuristic evaluation will be conducted with 5‐8 HIV experts. Eligible participants will have professional experience as an HIV counselor, researcher, health practitioner (eg, HIV care providers), pharmacist, and/or social and community health worker. After providing online consent, participants will complete an online demographic questionnaire followed by the chatbot response evaluation questionnaire. The demographic questionnaire consists of 6 deidentified questions asking participants about their age, gender, ethnicity, race, region, professional title, years of experience as an HIV expert, and frequency of interaction with patients. The evaluation questionnaire will use a 5-point Likert scale to assess 10 evaluation criteria (Table 1) for 6 pairs of user queries and chatbot responses. Participants will have the opportunity to provide some additional information and human sample responses to help improve chatbot responses.

To measure the consistency of ratings provided by the experts, we will calculate the intraclass correlation coefficient (ICC) [101] for the ratings of each criterion. We will then compare the chatbot-generated responses to expert responses by measuring Bilingual Evaluation Understudy (BLEU; IBM researchers Kishore Papineni, Salim Roukos, Todd Ward, and Wei-Jing Zhu) [89] and Recall-Oriented Understudy for Gisting Evaluation - Longest Common Subsequence (ROUGE-L; Chin-Yew Lin) [102] scores. These scores computationally measure relevance and linguistic similarity, and are scalable for evaluating larger datasets. In addition, human emotions are complex and are influenced by individual perception; this makes generating text for personalized emotional support a highly challenging endeavor.

Results and analysis of expert opinions will be published in a subsequent paper.

#### Future Large-Scale User Evaluation

To evaluate the system’s real-world utility, we will conduct a large-scale user study involving 50 PrEP candidates. Participants will be recruited in coordination with HIV prevention experts and established PrEP programs, using public resources such as referral lists, health websites, and online directories. All study procedures will be conducted in accordance with institutional review board (IRB) standards, with recruitment facilitated through IRB-approved flyers and digital outreach materials.

Eligibility criteria include that participants must be at least 18 years of age, proficient in English, and meet clinical eligibility for PrEP. Following the provision of informed online consent, participants will complete a series of comprehensive study questionnaires followed by a concluding interview. The estimated time commitment for the session is between 90 and 120 minutes.

To ensure methodological consistency across evaluation phases, this user study will use the same 10-point evaluation criteria and questionnaires used in the exploratory user evaluation. By incorporating qualitative measures (eg, semistructured interviews), we aim to gain deeper insights into user experience, perceived trust, and the effectiveness of the chatbot-provided PrEP support. This will allow us to assess how the chatbot performs in real-world scenarios beyond research settings.

#### Ethical Considerations

##### Institutional Approval

The exploratory user evaluation was reviewed and approved by the IRB at the University of North Carolina at Charlotte (protocol number IRB-26‐0423). The study was determined to pose minimal risk to participants, as the data collection was limited to subjective evaluations of chatbot responses and did not collect any personally identifiable information. For the planned heuristic evaluation and subsequent large-scale user study, separate IRB applications will be submitted for approval prior to recruitment, ensuring all ethical standards are met for that specific population.

##### Participant Consent and Incentives

Informed consent is obtained electronically via DocuSign (DocuSign Inc Team) from all participants, including students, community members, and HIV experts, prior to study commencement. Participants are informed about the study details, potential risks, participation rights, and incentives. They are explicitly informed that their participation was voluntary and that they could withdraw at any time.

In the exploratory user evaluation, students received the opportunity to replace one class lab activity as an incentive, while other participants received no compensation; an alternative nonresearch activity was available to students to ensure the voluntary nature of participation. Participants in the expert and large-scale user study will receive a US $25 Amazon gift card on completion of the study, subject to IRB approval.

##### Privacy and Data Security

To protect participant privacy, all data were or will be collected and analyzed anonymously, ensuring that no feedback could be traced back to individual experts or professionals. The dataset is filtered to pseudonymize any PII information such as names, email addresses, contact information, and IP addresses during the RAG process.

## Results

### Overview

We have developed a RAG chatbot prototype as of January 2025 and have iteratively refined the chatbot design and system based on feedback from internal evaluation.

### Support Classifier Performance

To assess the reliability of the RAG chatbot in identifying support needs, we validated the 3-class support classifier in categorizing queries into informational, emotional, and contextual needs.

As reported in Tables 2 and 3, our fine-tuned Gemma 2 2B-it model demonstrated strong performance in classifying PrEP support needs when fine-tuned on the full dataset (*F*_1_-score=0.86) and class-balanced subset (*F*_1_-score=0.89). Specifically, we found that fine-tuning the Gemma 2 2B-it model on a class-balanced subset indicated strong accuracy and precision in classifying emotional needs, while maintaining comparable performance in classifying informational and contextual queries (Table 2). Based on superior performance indicated by *F*_1_-score metrics, we used the class-balanced Gemma 2 2B-it model during preprocessing for classifying user support needs. In the context of classifying informational and emotional support seeking, our class-unbalanced BERT model exhibits superior performance (*F*_1_-information score=0.92, *F*_1_-emotion score=0.71) compared to a previous social support seeking dataset, CHQ-SocioEmo (*F*_1_-information score=0.58, *F*_1_-emotion score=0.62). This classification of user queries allows our chatbot to identify user needs, extract context, and generate tailored and emotionally relevant responses.

**Table 2.** Results of an assessment of the performance of 3 classifiers, fine-tuned on the class-balanced PrEP^a^ dataset. These classifiers are used for classifying user input into 3 classes.

Class	BERT-base-uncased^b^		Gemma 2B-it		Gemma 2 2B-it	
	*F*_1_-score	Accuracy	*F*_1_-score	Accuracy	*F*_1_-score	Accuracy
Information	0.93	0.94	0.92	0.88	0.93	0.90
Emotion	0.85	0.88	0.86	0.88	0.87	0.90
Context	0.85	0.82	0.86	0.88	0.86	0.86
Macro average	0.88	0.88	0.88	0.88	0.89^c^	0.89^c^

a PrEP: preexposure prophylaxis.

b BERT: Bidirectional Encoder Representations from Transformers.

c Best-performing model.

**Table 3.** Results of an assessment of the performance of 3 classifiers fine-tuned on the full PrEP^a^ dataset. These classifiers are used for classifying user inputs into 3 classes.

Class	BERT-base-uncased^b^		Gemma 2B-it		Gemma 2 2B-it	
	*F*_1_-score	Accuracy	*F*_1_-score	Accuracy	*F*_1_-score	Accuracy
Information	0.92	0.929	0.92	0.927	0.92	0.92
Emotion^c^	0.71	0.693	0.69	0.624	0.71	0.663
Context	0.95	0.948	0.95	0.964	0.96	0.966
Weighted average	0.92	0.92	0.92	0.922	0.92	0.93^d^

a PrEP: preexposure prophylaxis.

b BERT: Bidirectional Encoder Representations from Transformers.

c Poor performance compared to class-balanced fine-tuned model predictions.

d Model with best performance.

### Prompt Engineering

We manually observed that prompt engineering plays a significant role in harnessing LLM capabilities to generate desired outputs. We report key findings from our iterative prompt engineering, offering suggestions for effective prompting in chatbot applications. Although there is no standard approach for refining prompts [103], starting with simple and direct instructions (Table S2 in Multimedia Appendix 1) helped in understanding LLM comprehension and reasoning ability. For complex and longer queries, we found that prompting the LLM to decompose the query into segments helps in improving the response comprehensiveness (eg, PI-2 prompt from Table 1). Such prompt decomposition was also effective in responding to subtle user emotions by first identifying the emotions from a list of emotion categories and guiding the LLM in social-emotional reciprocation for personalizing emotional responses (ie, PE-1 and PE-2 prompts).

An important finding in LLM prompt engineering was the limitation of using multiple instructions in a single prompt. We found that LLMs indicate low prompt adherence when a prompt has multiple instructions. In such cases, the LLM often defaults to the last instruction, resulting in partial and inconsistent execution of complex logical reasoning prompts. Another challenge was the LLM’s sensitivity to linguistic variability in prompts [104]. We found that paraphrasing prompts by modifying prompt structure, using synonyms, and expanding prompt context is ineffective in context management tasks (eg, identifying the need for additional context), exhibiting inconsistent response output. We found that shorter prompts with content words summarizing the main concept or idea can reduce ambiguity in LLM decisions.

A few-shot prompting approach is effective in text classification [49]. We found that few-shot prompting did not consistently perform well across classification for informational needs, emotional needs, and context. This limitation is potentially because these categories represent high-level support needs that require diverse perspectives for inferring ambiguous context and broad scope. Consequently, fine-tuning LLMs on annotated datasets produced more reliable results than few-shot prompting. Few-shot prompting is effective in simple subclassification tasks with clear and distinct criteria for narrow scope [105].

### Fact Validation

For fact validation, deterministic techniques, including semantic relevance and text similarity metrics (eg, ROUGE and BLEU), often failed due to their limited ability to capture negations [106] and numerical accuracy [107]. We found that LLMs generally struggle to validate statements that are generalized or personalized and are not directly present in the knowledge base. When required facts are scattered across documents, such generalized or personalized statements need implicit validation and cross-referencing for accurate verification of information [108].

### Exploratory User Study Analysis

The exploratory user study included 7 participants who completed the full evaluation protocol; the majority of participants were female (5/7, 71.4%) and aged 25‐34 years (5/7, 71.4%), with an Asian (5/7, 71.4%) ethnic background. Across 15 real-world queries evaluated against 10 quality criteria, the study generated 3144 valid observations, with 6 “NA” ratings (instances where a criterion was deemed nonapplicable by the rater).

### Preliminary Comparison of RAG Responses With General LLM and Real-World User Responses

The overall performance of the 3 response sets (general LLM, RAG chatbot, and user) was evaluated across 10 distinct evaluation criteria. Due to nonindependence of data points, a linear mixed model was used to account for user bias and random effects from both participants and specific questions.

Initial observations indicate variations in response quality across the 3 groups, suggesting specific areas for improvement in the RAG chatbot architecture and prompt design based on the evaluation criteria. Both AI modalities (general LLM and RAG) had higher preference across the evaluation criteria compared to user responses. We observed that the general LLM’s communication style appeared to influence how participants rated comprehensiveness, detail, and tailoredness of the information received, though both AI models showed similar performance in terms of empathy and tone.

Given the exploratory nature of this pilot evaluation, the sample size (n=7) was small and skewed in terms of participant demographics (eg, primarily consisting of university students), which limits the generalizability of these findings to the broader PrEP-candidate population. However, these exploratory insights into future modifications demonstrate the viability of the evaluation framework and provide a descriptive baseline for the heuristic and user study outlined in this protocol.

## Discussion

### Principal Findings

We describe the development of a RAG chatbot prototype for providing personalized support to fulfill informational, peer experiential expertise, and emotional needs of PrEP candidates to facilitate PrEP care. The chatbot is designed for (1) identifying support needs and personalizing responses (tailored information, clarity, and tone); (2) providing complete, relevant, and accurate HIV and PrEP information (comprehensiveness, detail, relevancy, accuracy, and language); (3) providing peer experiential expertise support (relevancy and actionability); and (4) responding with human-like emotional support (empathy).

### Performance Gap of AI Modalities

Our exploratory user study results provide initial empirical support for the proposed chatbot functionality. Both the RAG chatbot and the general LLM were preferred over user responses across all 10 evaluation criteria. This suggests that AI-generated support can offer an enhanced level of perceived utility, informational depth, and structural coherence compared to traditional peer support related to PrEP.

### Addressing Technical Challenges

The analysis revealed a performance gap between the two AI architectures. The general LLM performed better than the RAG chatbot in 8 of 10 categories, with the exceptions of “empathy” and “tone.” While RAG techniques are intended to reduce hallucinations [48], improve content diversity [58], user privacy [53], and personalization [54,55], our results indicate that the logical reconstruction of fragmented information remains a challenge for RAG-based systems [108].

LLMs can maintain superior structural fluidity and generate well-organized information [109]. Our results indicated higher ratings for the general LLM in stylistic criteria, such as structure and communication style. In contrast, we observed that the RAG system’s output was inherently tied to multisource retrieval and the informal nature of the retrieved peer-discussion data. However, the practical advantage of our RAG approach lies in its modular architecture, which is specifically designed to facilitate control over response accuracy and prevent the generation of fictitious experiences by relying on verifiable information. Furthermore, our RAG chatbot integrates anonymized real-world peer experiential expertise and Willcox emotional vocabulary to provide a level of cultural authenticity and empathetic alignment that a general-purpose model cannot guarantee. Another advantage of RAG models is their ability to use smaller, localized language models to achieve response quality comparable to significantly larger LLMs [110,111], thereby providing opportunities to reduce health care barriers related to data privacy and operational costs.

The participant group expressed a preference for the structured and categorized communication style of the general LLM over the RAG chatbot’s response format. This suggests that while RAG can be highly effective in extracting information from diverse sources to provide contextually intelligent responses [112], further refinement is needed to synthesize retrieved fragments into a clearer and more structured response format that matches the systematic response delivery of general-purpose LLMs.

### User Fatigue and Rater Consistency

Our analysis revealed a residual variance of 0.662 within the linear mixed model, suggesting random noise within the rater data that warrants further investigation. We believe that this inconsistency is primarily attributable to user fatigue stemming from the study’s cognitive load. Each participant was required to evaluate 15 complex query sets across 10 distinct criteria, resulting in 450 total data points per rater.

Furthermore, the high level of domain-specific expertise required to evaluate PrEP support, specifically the distinction between clinical accuracy and nuanced peer experiential support, may have been impacted by the nonexpert and nontarget user status of the participant group. As the raters were neither clinicians nor PrEP candidates, their evaluations of criteria such as “accuracy,” “actionability,” “clarity,” “comprehensiveness,” “detail,” “empathetic,” “language,” “relevance,” “tailored information,” and “tone” represent perceived credibility rather than objective medical validation. For criteria related to information quality (accuracy, clarity, actionability, and detail), raters lacked the clinical training required to verify the medical correctness of PrEP information and practical guidance. Similarly, evaluating criteria on response relevance and appropriateness (relevance, tailored information, and comprehensiveness) requires an understanding of PrEP challenges such as medical mistrust, PrEP navigation, or side-effect management, commonly experienced by the target population. Furthermore, HIV experts and PrEP candidates are more familiar with PrEP terminology and community lingo, which may impact the perception of tailored information, language, and trust, as these target users can distinguish between generic AI responses and grounded, community-informed experiential support.

The low incidence of “N/A” ratings (n=6) suggests that the questionnaire structure may have lacked sufficient clarity in guiding participants to opt out when specific criteria were not relevant to a particular question sample. Improving the questionnaire structure to better highlight the specific intent of each rating and providing more robust instructions will be critical to ensure rater reliability. These exploratory observations will directly inform the next phases of our development, as detailed in the “Future Direction” section.

### Implications

#### Overview

RAG chatbots are increasingly used to provide various support for health care use and decision-making [56,113-116]. We report the first published efforts to develop a RAG chatbot prototype for providing personalized information, peer experiential expertise, and emotional support to PrEP candidates. Our chatbot aims to enhance PrEP support by leveraging verified information, real-world experiences, and emotional considerations. It can also interpret complex context-dependent queries, and through personalized informational, peer experiential expertise, and human-like emotional support, we believe this approach has the potential to clarify misconceptions about PrEP and HIV treatment, support user confidence, and facilitate future PrEP uptake.

#### Data Privacy and Ethical Implications

The RAG chatbot currently leverages knowledge from Reddit, with a future plan to expand to include other online platforms. All data usage complies with the respective platforms’ user agreements and policies, accessed in February 2024, and the facts dataset, collected in January 2022. Recognizing the sensitive nature of health-related discourse in these communities, our data handling process incorporates privacy safeguards; we do not attempt to identify users and protect privacy by filtering personally identifiable information (eg, name, email addresses, IP addresses, and specific unique identifiers).

The proposed RAG architecture is designed to enhance response precision; however, the deployment of personalized PrEP chatbots carries inherent ethical implications. A primary concern is the probabilistic nature of LLMs. Despite the integration of domain-specific knowledge, these models remain prone to hallucination [48,49] and may lack the deep contextual understanding required for nuanced medical inquiries. Consequently, there is a risk of users overrelying on the tool [117,118] for definitive medical advice, which could delay professional clinical consultation.

Furthermore, it is critical to distinguish between the cognitive empathy simulated by the LLM and true human-like emotional intelligence. While the system can identify and mirror user sentiments, it lacks the capacity for genuine affective responses [43]. This limitation is particularly significant in the context of HIV and PrEP, where users may present with complex psychological distress. The chatbot responses could inadvertently lead to stigma reinforcement if the model misinterprets nuanced emotions and sensitive disclosures or provides inappropriate feedback [117,118].

To mitigate these ethical concerns, the RAG architecture implements a modular data governance strategy. The chatbot is designed to use social media data exclusively for addressing experiential queries and mirroring human-like empathetic communication styles while being guided by the Willcox Feeling Wheel to interpret user emotions. Conversely, all factual and clinical queries are strictly routed to the manually curated factual database, ensuring that health-related information is derived solely from verified public health organizations. For transparency, any user content referenced in chatbot responses is quoted anonymously to mitigate the risk of reidentification while maintaining the authenticity of the peer-to-peer perspective.

### Limitations

Although recent advances in hallucination removal have improved LLM reliability, these advances do not entirely eliminate the need for validating response accuracy, especially in health care applications [92]. Due to linguistic complexity and nuances in generated responses, LLMs struggle to consistently and accurately validate factual information using only RAGs [119]. Additional methodological improvements are needed to validate broad facts outside of the database and context-dependent information.

Our system faces several inherent risks and ethical considerations. First, the complexity of human emotion makes personalized digital support a challenging endeavor, with risks of stigma reinforcement or user overreliance for clinical advice. Second, while using community-sourced data (eg, Reddit) is practical, it may introduce demographic or topical biases that limit the system’s generalizability across diverse PrEP user populations. We intend to extend the datasets from MedHelp and POZ communities to mitigate these gaps.

A limitation of this study is the absence of a comprehensive quantitative analysis comparing the performance of few-shot prompting and fine-tuning for support classification. While our study provides initial qualitative insights into the effectiveness of these 2 approaches for specific tasks, these findings are exploratory. Future work should include extensive empirical comparisons to statistically generalize these results across broader datasets.

Another limitation of the current evaluation is that the user study was conducted with people who were nontarget PrEP-using demographics and nonclinical experts. While this provides insight into general user perception and readability, it may not capture the technical accuracy or perceived personalization of the responses with precision. Future work will include larger-scale iterative evaluations to identify real-world failure scenarios and ensure the model’s safety profile. Furthermore, subsequent iterations will focus on demographic-aware personalization to better reflect the diverse cultural preferences, behaviors, and potential linguistic needs of the PrEP users, addressing the wider ethical implications of using sensitive, public-domain social media data for health support.

### Future Direction

The current chatbot model was developed and accessed in a research setting and will be rigorously tested for safety and reliability with experts and the target population prior to real-world deployment. Building on our exploratory findings, we will conduct a larger-scale user study for gathering feedback on chatbot responses from the target population. We will also concurrently initiate a heuristic evaluation with HIV prevention experts to validate clinical safety, user trust, ethical alignment, and factual accuracy of the PrEP chatbot.

To bridge the gap between initial research product and sustainable real-world health intervention, the chatbot design will incorporate sociotechnical design considerations [120] through the Accelerated Creation-to-Sustainment (ACS) framework [121]. Through these community-representative user and expert evaluations, we will gather feedback on cultural and behavioral preferences to improve long-term adoption and user trust. This includes incorporating multilingual support to cater to linguistic needs. Furthermore, we will evaluate technological flexibility, including low-cost, lightweight local deployment models and multiplatform accessibility (eg, mobile and web-based interfaces), to lower entry barriers in resource-limited settings. To ensure long-term sustainability, we plan to engage with HIV health stakeholders to establish frameworks for managing scalability and routine content updates, ensuring the intervention remains clinically accurate and relevant as the HIV prevention landscape evolves.

### Conclusion

This study details the implementation of a RAG chatbot prototype designed to provide personalized informational, peer experiential expertise, and emotional support to PrEP candidates. By offering personalized information and peer experience, our chatbot can provide essential information for making informed decisions about health. In addition, our chatbot’s capability to provide human-like emotional support highlights its potential to address stigma related to PrEP. Although current observations are preliminary, we believe our chatbot has the potential to clarify misconceptions and provide PrEP support through these mechanisms. Future evaluation using expert and real-world feedback will validate and improve the chatbot’s capability; after this, a trial to demonstrate the efficacy of the tool to increase PrEP uptake and retention will be proposed.

## Supplementary material

10.2196/79966 Multimedia Appendix 1 Prompt engineering.

## Abbreviations

ACS: Accelerated Creation-to-Sustainment
BERT: Bidirectional Encoder Representations from Transformers
BLEU: Bilingual Evaluation Understudy
CN-1: context handling prompt 1
CN-2: context handling prompt 2
CR: cohesive response
EHE: Ending the HIV Epidemic
IC: information subclassification
ICC: intraclass correlation coefficient
IRB: institutional review board
LLM: large language model
LoRA: Low-Rank Adaptation
PE: personalized emotion
PEFT: Parameter-Efficient Fine-Tuning
PI: personalized information
PrEP: preexposure prophylaxis
PX: personalized experience
RAG: retrieval-augmented generation
ROUGE-L: Recall-Oriented Understudy for Gisting Evaluation - Longest Common Subsequence
SAT: Segment Any Text
SBERT: Sentence-Bidirectional Encoder Representations from Transformers
SSBC: Social Support Behavior Code
