# Supplementary material for: A Conversational Agent for Providing Personalized Preexposure Prophylaxis (PrEP) Support: Protocol for Chatbot Implementation and Evaluation
Source: JMIR Res Protoc. 2026 Oct 1;15:e79966. doi: 10.2196/79966 (PMC13630282; doi:10.2196/79966)
Supplement: Supplementary file 1 [file resprot-v15-e79966-s001.docx]

**Prompt Engineering**

| ***Table S1.*** *Refined prompts used for providing personalized informational, peer experiential expertise, and human-like emotional support.* | | |
| --- | --- | --- |
| **Category** | **Description** | **Prompt** |
| ***Context Handling (CN) Prompts*** | CN-1: Prompt used for identifying whether additional context is needed | [{query}]  Identify whether the query enclosed in square brackets above is self-contained. Provide corresponding one-word response as 'Yes' if self-contained and 'No' if not self-contained. |
|  | CN-2: Prompt used for instructing LLM to ask user for additional context | Ask user to provide more information or context in relation to the query enclosed in square brackets. [{query}] |
| ***Information Subclassification (IC) Prompt*** | IC: Prompt for sub-classifying information queries as seeking information or peer experiential expertise support | Classify the following text as seeking advice, experience, or information: Text: should i take this medicine. Classification: Advice Text: Looking for support, personal stories, or experiences. Classification: Experience Text: what is PrEP Classification: Information Text: 'I don't know what to do. Classification: Advice Text: How are other people coping with PrEP side effects? Classification: Experience Text: Is it worth to buy Truvada?. Classification: Information  Text: |
| ***Personalized Information (PI) Prompts*** | PI-1: Prompt used for identifying topic discussed in the query for retrieving similar documents based on topic match. | [{context} {query}] Topic list = {topic_list} From the above topic list, assign a single main topic that is most suitable to the text enclosed in square brackets. If none of the topics is most suitable, review the above topic list and pick a closely related topic. Provide only the topic. |
|  | PI-2: Prompt used for generating responses to informational query using the retrieved documents. | [{messages}] Segment the following query and provide a detailed response to all segments in paragraphs by using **only the information enclosed in square brackets above. Provide a single response combining all segment responses in plain paragraph format and **do not specify the segments. Also, **Remember not to use unnecessary special characters in the response. - {context} {query} **Important instruction: Generate a response without containing sentences with phrases like 'the provided text', 'the passage', 'the document', 'the information', 'the text', 'provided information', or 'provided text'. |
|  | PI-3: Prompt used to refine generated response. | [Query: {context}{query}  Response: {response text}]  For the user query and response pair enclosed in square brackets, check if the response is consistent with the query in details such as location, age, and gender. If the response is not consistent, paraphrase the response to ensure that the response is general and does not include any details related to location, age, gender. **Only provide the paraphrased response. If the response is consistent provide **only the response text without the prefix Response: and without the square brackets. |
| ***Personalized Experience (PX) Prompt*** | Prompt used for generating response to patient expertise query using the retrieved documents. | Using **only the information enclosed in square brackets above, provide a response to the following query - {context}{query} Present the response as a third person narrative opinion, starting with something as "There are people with similar experience ....". You can also give direct speech example of the user experience. **Remember to end the response stating that the information in the response is based on other people's experience. **Important instruction: Generate a response without containing sentences with phrases like 'the provided text', 'the passage', 'the document', 'the information', 'the text', 'provided information', or 'provided text'. |
| ***Personalized Emotional (PE) Prompts*** | PE-1: Prompt used for generating response to emotional query using the retrieved documents. | Consider the following Feeling = Emotion categories: Sad = Tired, bored, lonely, depressed, ashamed, guilty, sleepy, apathetic, isolated, inferior, stupid, remorseful Mad = Hurt, hostility, angry, selfish, hateful, critical, distant, sarcastic, frustrated, jealous, irritated, skeptical Scared = Confused, rejected, helpless, submissive, insecure, anxious, bewildered, discouraged, insignificant, inadequate, embarrassed, overwhelmed Joyful = Excited, sensuous, energetic, cheerful, creative, hopeful, daring, fascinating, stimulating, amused, playful, optimistic Powerful = Aware, proud, respected, appreciated, important, faithful, surprised, successful, worthwhile, valuable, discerning, confident Peaceful = Nurturing, trusting, loving, intimate, thoughtful, content, thankful, secure, serene, responsive, pensive, relaxed  Identify the feeling expressed in the following expression based on the feelings=emotions listed above - {query}  For the identified feeling, generate a suitable response providing understanding, empathy, sympathy, reassurance, validation, relief of blame, or compliment, for the following user query - {context}{query}. You can also use any emotional context from the information enclosed in square brackets above to support your response. Do not use facts from the information enclosed in square brackets above to support your response. Only provide the final response without the identified feeling. Also, **Do not use unnecessary special characters in the final response. |
|  | PE-2: Prompt used for generating responses to emotional query when no relevant documents are retrieved. | Consider the following Feeling = Emotion categories: Sad = Tired, bored, lonely, depressed, ashamed, guilty, sleepy, apathetic, isolated, inferior, stupid, remorseful Mad = Hurt, hostility, angry, selfish, hateful, critical, distant, sarcastic, frustrated, jealous, irritated, skeptical Scared = Confused, rejected, helpless, submissive, insecure, anxious, bewildered, discouraged, insignificant, inadequate, embarrassed, overwhelmed Joyful = Excited, sensuous, energetic, cheerful, creative, hopeful, daring, fascinating, stimulating, amused, playful, optimistic Powerful = Aware, proud, respected, appreciated, important, faithful, surprised, successful, worthwhile, valuable, discerning, confident Peaceful = Nurturing, trusting, loving, intimate, thoughtful, content, thankful, secure, serene, responsive, pensive, relaxed  Identify the feeling expressed in the following expression based on the feelings=emotions listed above - {query}  For the identified feeling, generate a suitable response providing understanding, empathy, sympathy, reassurance, validation, relief of blame, or compliment, for the following user query - {context}{query}. Only provide the final response without the identified feeling. Also, **Do not use unnecessary special characters in the final response. |
| ***Fact Validation (FV) Prompt*** | FV-1: Prompt used for verifying fact consistency. | Review the following list of response and document sentence pairs and identify responses that are inconsistent with the corresponding document sentences. Only provide the inconsistent response sentences with their key and value as a list of json dictionary. If all response-doc pairs are consistent, say 'All consistent'.  Consistency means that all information provided in the response text is fully supported by the document text. If the response text contradicts, differs or provides partially incorrect information with respect to the document text, it is inconsistent. Start response as Response = {fact_list} |
|  | FV-2: Prompt used for extracting supporting facts for inaccurate response-document pairs. | Text: [{text}]  For each of the response sentences in the json list below, extract corresponding sentences from the text enclosed in square brackets above, that fully support the specific response sentence. If no supportive sentence is found, replace 'document_sentence' value as "" else replace the 'document_sentence’ value with the extracted text in the json dictionary. Only provide the json response starting as 'json []' without additional text.  Return **only** a valid JSON response:  ```json  {{      "id": "0",      "response_sentence": "...",      "document_sentence": "...",  }}  {answer} |
| ***Cohesive Response (CR) Prompt*** | Prompt used for combining responses generated for sub-queries. | [{response text}]  Smoothen the flow of information in the text enclosed in square brackets. Only provide the final response. |

| ***Table S2.*** *Initial prompts used for generating LLM responses to provide personalized PrEP support. These prompts were iteratively refined by developing several intermediate prompts to improve the quality of generated responses.* | | |
| --- | --- | --- |
| **Category** | **Description** | **Prompt** |
| ***Context Handling (CN) Prompts*** | CN-1: Prompt used for identifying whether additional context is needed | Classify whether the user needs to provide any additional information or context for you to answer the following query - {query} |
|  | CN-2: Prompt used for instructing LLM to ask user for additional context | Ask user to provide more information or context in relation to the query enclosed in square brackets. [{query}] |
| ***Information Subclassification (IC) Prompt*** | IC: Prompt for sub-classifying information queries as seeking information or experiential expertise support | Classify the following text as seeking advice, experience, or information: {query} |
| ***Personalized Information (PI) Prompts*** | PI-1: Prompt used for identifying topic discussed in the query for retrieving similar documents based on topic match. | Topic list = {topic_list} From the above topic list, identify a single main topic that is discussed in the following query, {query}. If does not directly relate identify a closely related topic. Provide only the topic. |
|  | PI-2: Prompt used for generating responses to informational query using the retrieved documents. | [{messages}] Using the information enclosed in square brackets, provide a response to the following user query. |
|  | PI-3: Prompt used to refine generated response. | [{response text}]  Consider the following user query – {query}. If the query does not contain specific details such as location, age, health condition, etc., check whether the response enclosed in square brackets contains such details and if it contains, paraphrase the text enclosed in square brackets to make sure that the response is general and does not include such details. Otherwise return the text enclosed in square brackets. |
| ***Personalized Experience (PX) Prompt*** | Prompt used for generating response to patient expertise query using the retrieved documents. | [{messages}]  Using the information enclosed in square brackets above, provide a response to the following user query |
| ***Personalized Emotional (PE) Prompts*** | PE-1: Prompt used for generating response to emotional query using the retrieved documents. | [{messages}]  Consider the following Feeling = Emotion categories: Sad = Tired, bored, lonely, depressed, ashamed, guilty, sleepy, apathetic, isolated, inferior, stupid, remorseful Mad = Hurt, hostility, angry, selfish, hateful, critical, distant, sarcastic, frustrated, jealous, irritated, skeptical Scared = Confused, rejected, helpless, submissive, insecure, anxious, bewildered, discouraged, insignificant, inadequate, embarrassed, overwhelmed Joyful = Excited, sensuous, energetic, cheerful, creative, hopeful, daring, fascinating, stimulating, amused, playful, optimistic Powerful = Aware, proud, respected, appreciated, important, faithful, surprised, successful, worthwhile, valuable, discerning, confident Peaceful = Nurturing, trusting, loving, intimate, thoughtful, content, thankful, secure, serene, responsive, pensive, relaxed  Identify the feeling expressed in the following expression based on the feelings=emotions listed above - {query}  For the identified feeling, generate a suitable response providing understanding, empathy, sympathy, reassurance, validation, relief of blame, or compliment. Use information from text enclosed in square brackets to generate an emotional response. |
|  | PE-2: Prompt used for generating responses to emotional query when no relevant documents are retrieved. | Consider the following Feeling = Emotion categories: Sad = Tired, bored, lonely, depressed, ashamed, guilty, sleepy, apathetic, isolated, inferior, stupid, remorseful Mad = Hurt, hostility, angry, selfish, hateful, critical, distant, sarcastic, frustrated, jealous, irritated, skeptical Scared = Confused, rejected, helpless, submissive, insecure, anxious, bewildered, discouraged, insignificant, inadequate, embarrassed, overwhelmed Joyful = Excited, sensuous, energetic, cheerful, creative, hopeful, daring, fascinating, stimulating, amused, playful, optimistic Powerful = Aware, proud, respected, appreciated, important, faithful, surprised, successful, worthwhile, valuable, discerning, confident Peaceful = Nurturing, trusting, loving, intimate, thoughtful, content, thankful, secure, serene, responsive, pensive, relaxed  Identify the feeling expressed in the following expression based on the feelings=emotions listed above - {query}  For the identified feeling, generate a suitable response providing understanding, empathy, sympathy, reassurance, validation, relief of blame, or compliment. |
| ***Cohesive Response (CR) Prompt*** | Prompt used for combining responses generated for sub-queries. | [{response text}]  Smoothen the flow of information in the text enclosed in square brackets. |
| ***Fact Validation (FV) Prompt*** | FV-1: Prompt used for verifying fact consistency. | Review the following list of response and document sentence pairs and identify responses that are inconsistent with the corresponding document sentences. Start response as Response =  {fact_list} |
|  | FV-2: Prompt used for extracting relevant facts and replacing inconsistent factual information. | Text: [{text}]  For each of the response sentences in the json list below, extract corresponding sentences from the text enclosed in square brackets above, that support the specific response sentence. If no supportive sentence is found, replace 'document_sentence' value as "" else replace the 'document_sentence’ value with the extracted text in the json dictionary. Only provide the json response starting as 'json []' without additional text.  Return **only** a valid JSON response:  ```json  {{      "id": "0",      "response_sentence": "...",      "document_sentence": "...",  }}  {answer} |
